# Immunosuppressive Effects of Natural α,β-Unsaturated Carbonyl-Based Compounds, and Their Analogs and Derivatives, on Immune Cells: A Review

**DOI:** 10.3389/fphar.2017.00022

**Published:** 2017-01-30

**Authors:** Laiba Arshad, Ibrahim Jantan, Syed Nasir Abbas Bukhari, Md. Areeful Haque

**Affiliations:** Drug and Herbal Research Centre, Faculty of Pharmacy, Universiti Kebangsaan MalaysiaKuala Lumpur, Malaysia

**Keywords:** α, β-unsaturated carbonyl-based compounds, curcumin, chalcone, zerumbone, immune cells, immunosuppressive effects

## Abstract

The immune system is complex and pervasive as it functions to prevent or limit infections in the human body. In a healthy organism, the immune system and the redox balance of immune cells maintain homeostasis within the body. The failure to maintain the balance may lead to impaired immune response and either over activity or abnormally low activity of the immune cells resulting in autoimmune or immune deficiency diseases. Compounds containing α,β-unsaturated carbonyl-based moieties are often reactive. The reactivity of these groups is responsible for their diverse pharmacological activities, and the most important and widely studied include the natural compounds curcumin, chalcone, and zerumbone. Numerous studies have revealed the mainly immunosuppressive and anti-inflammatory activities of the aforesaid compounds. This review highlights the specific immunosuppressive effects of these natural α,β-unsaturated carbonyl-based compounds, and their analogs and derivatives on different types of immune cells of the innate (granulocytes, monocytes, macrophages, and dendritic cells) and adaptive (T cells, B cells, and natural killer cells) immune systems. The inhibitory effects of these compounds have been comprehensively studied on neutrophils, monocytes and macrophages but their effects on T cells, B cells, natural killer cells, and dendritic cells have not been well investigated. It is of paramount importance to continue generating experimental data on the mechanisms of action of α,β-unsaturated carbonyl-based compounds on immune cells to provide useful information for ensuing research to discover new immunomodulating agents.

## Introduction

The immune system is the body's complex and pervasive defense system which maintained the integrity of the body and protects it from microbial invasion. It refers to organization of immune cells and specialized immune molecules that have evolved to mediate resistance to infections. The immune system can be categorized into two major divisions i.e., innate immune system and adaptive immune system. The innate immune system refers to non-specific immunity as it is destined to attack any foreign particle invading the body and hence serve as primary defense for the body. Neutrophils, macrophages, and dendritic cells are the central players of innate immunity. Contrary to this, adaptive or acquired immunity produces a specific effect in response to an antigen and reserves the feature of immunological memory that distinguishes it from the aforementioned response. The members of acquired immunity involve majorly T cells and B cells (Lee et al., 2015). In healthy organism, the immune system and the adequate redox balance of immune cells maintain homeostasis within the body. The failure to maintain the balance may lead to impaired immune response and hence an abnormal immune system (Ilangkovan et al., 2015). Most of the immune system disorders cause abnormally low activity or over activity of the immune cells. In cases of immune system over activity, the body attacks and damages its own tissues referring to acquired immune system reaction (autoimmune diseases) while immune deficiency diseases decrease the body's ability to fight invaders, causing vulnerability to infections. Among the most common diseases are rheumatoid arthritis, type1 diabetes, systemic lupus erythematosus, tuberculosis, and atherogenesis. The autoimmune destruction of the pancreatic β cells within the islets of Langerhans results in the progression of type 1 diabetes (Mehrotra et al., 2013).

The word immunomodulation circles around the concept of reorganizing the immune system to achieve the therapeutic benefit. The aim of immunomodulators or immunotherapy is to modify the immune response according to pathological condition. Generally, there are two clinical situations when the immune system are required to be managed i.e., when the immune response is abnormally decreased (immunodeficiency) e.g., cancer, hepatitis, HIV and tuberculosis or increased like diabetes, arthritis, lupus, eczema, and multiple sclerosis (autoimmunity). In case of immunodeficiency, a therapy that particularly boosts up the immune response called immunostimulants is desired while immunosuppressant abolished the overactive immune system. Augmentation of the immune response is desirable to prevent infection in states of immunodeficiency or in case of overactive immune system. Immunoadjuvants is another term used nowadays, are specific immune stimulators which enhance the efficacy of vaccine (Gea-Banacloche, 2006). Over many decades, many natural, or synthetic immunomodulators are being used for managing immune disorders. This review is to provide updated overview of specific immunomodulatory effects of the most important and widely studied natural products containing α,β-unsaturated carbonyls, i.e., curcumin **(1)**, chalcone **(2)**, and zerumbone **(3)**. The activities of these natural products and their analogs and derivatives on different types of immune cells of the innate and adaptive immune systems have been comprehensively discussed.

## Natural α,β-unsaturated carbonyl-based compounds

**Figure 1:**
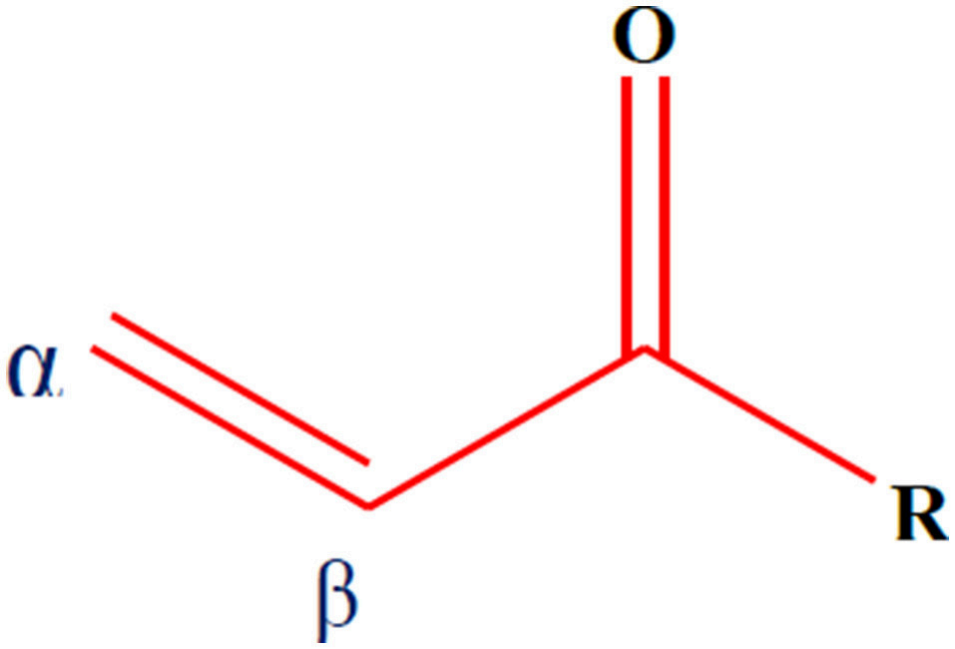
**General structure of α,β-unsaturated carbonyl-based compounds**.

**Figure 2:**
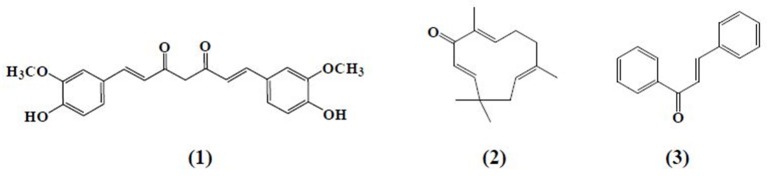
**Chemical structures of natural α,β-unsaturated carbonyl-based compounds**.

α,β-Unsaturated carbonyl-based compounds refers to such structures possessing α,β-carbonyl moieties. In such compounds the carbon-carbon double bond and carbon-oxygen double bond are segregated by only one carbon-carbon single bond i.e., double bonds are conjugated. These conjugations not only give the properties of the functional group attached but also give some other properties to the compounds. Figure 1 illustrates the general structure of α,β-unsaturated carbonyl-based compounds. These compounds are considered to be the most reactive substructures of natural products or synthetic molecules. The reactivity of these groups owes a diverse array of pharmacological activities. The most important and widely studied α,β-unsaturated carbonyl-based compounds include the natural products, curcumin **(1)**, zerumbone (**2**), chalcone **(3)**, and their analogs and derivatives. The structures of curcumin, chalcone, and zerumbone are shown in Figure 2. In fact one-sixth of the currently known natural products contain these potentially reactive substructures (Rodrigues et al., 2016).

Curcumin **(1)**, the active principle of turmeric, is a hydrophobic polyphenol mainly isolated from the rhizomes (turmeric) of *Curcuma longa* (Anand et al., 2008). Chemically, it is a bis-α,β-unsaturated β-diketone composed of two aromatic rings joined together by two carbonyl groups. Despite the fact that curcumin is a multi-targeting agent and safer at higher doses, poor bioavailability, and solubility is still a point at issue for the researchers. To overcome this problem, scientists adopted a number of strategies including formulation of curcumin with adjuvant like piperine, curcumin-based drug delivery system, and structural modification of curcumin (Anand et al., 2007). These approaches have not yet achieved the desired therapeutic purpose but somewhat improved the pharmacokinetic profile of curcumin. The curcumin analogs were synthesized in a number of different ways either preserving the β-diketone moiety or removing it. Previous studies reported different laboratory methods involving multiple types of catalysts for the synthesis of curcumin-like structures but the most common method was found to be a Claisen–Schmidt condensation reaction, between a ketone and aldehyde in the presence of a polar solvent (Anand et al., 2008).

Zerumbone **(2)**, (2E,6E,10E)-2,6,9,9-tetramethylcycloundeca-2,6,10-trien-1-one, a crystalline monocyclic sesquiterpene, is mainly isolated from the rhizomes of shampoo ginger (*Zingiber zerumbet*). Zerumbone was isolated for the first time in 1960 and structurally elucidated in 1965 (Dev, 1960; Damodaran and Dev, 1965). It contains α,β-unsaturated carbonyl moiety along with 3 double bonds, one at C-2 and the rest at C-6 and C-9 positions. Chalcone **(3)** is a member of flavonoid family with a 1, 3-diphenyl-2-propen-1-one skeleton. Analogous to curcumin, the structure of chalcone consists of two aromatic rings linked by a three carbon α,β-unsaturated carbonyl system. Chalcone and chalcone-based derivatives can be obtained using variety of laboratory methods and schemes. Numerous lines of evidence suggest that acid or base catalyzed aldol condensation or Claisen–Schmidt condensation of acetophenones with aromatic aldehydes are among the most conventional methods (Datta et al., 1971; Makrandi and Kumar, 2004).

Previous studies have indicated that these natural α,β-unsaturated carbonyl compounds and their analogs and derivatives exhibited a wide range of biological activities. A number of studies have showed that zerumbone, chalcone derivatives and curcumin analogs exerted immunomodulatory role (Feng et al., 2005; Magerramov et al., 2006; Keong et al., 2010; Bukhari et al., 2012, 2013b, 2014a; Lal et al., 2012). Likewise, antioxidant, anti-inflammatory, antiviral, antibacterial, antifungal, and antitubercular activities have been exhibited by these compounds (Lin et al., 2002; Murakami et al., 2002, 2004; Batovska et al., 2007; Sivakumar et al., 2007; Tomar et al., 2007; Trivedi et al., 2007; Chiaradia et al., 2008; Chien et al., 2008; Sulaiman et al., 2010; Jantan et al., 2012; Du et al., 2013; Ou et al., 2013; Bukhari et al., 2013a; Ahmad et al., 2014; Chen et al., 2014; Goodarzi et al., 2014). Recently, some studies reported that α,β-unsaturated moiety displayed acetylcholine esterase (AChE) and butylcholine esterase (BuChE) inhibition and may serve as promising agent in the treatment of alzeihmer's disease (Bag et al., 2013; Silva et al., 2013; Bukhari et al., 2014b). In addition, the ketone functional group of these compounds is believed to possess anticancer potential (Sahu et al., 2012; Bukhari et al., 2014c; Qin et al., 2016).

Recently, Rodrigues et al. (2016) suggested that compounds containing α,β-unsaturated carbonyls may not serve as good leads for drug design because of their potential to undergo Michael addition reaction leading to unwanted side effects, such as cytotoxicity and cell-damage. However, direct interaction of the groups with enzymes can be desirable. For example these compounds with Michael-accepting features can block carcinogenesis by inducing certain enzymes such as glutathione S-transferase and quinone reductase activity, which inhibits the reactive electrophilic forms of carcinogens. α,β-Unsaturated carbonyls may also be desirable moieties as they also have the ability to be radical scavengers through covalent ligand binding to target proteins or act as antioxidants, for example by thiol trapping.

## Immunosuppression of natural α,β-unsaturated carbonyl-based compounds on the innate immune system

**Figure 3:**
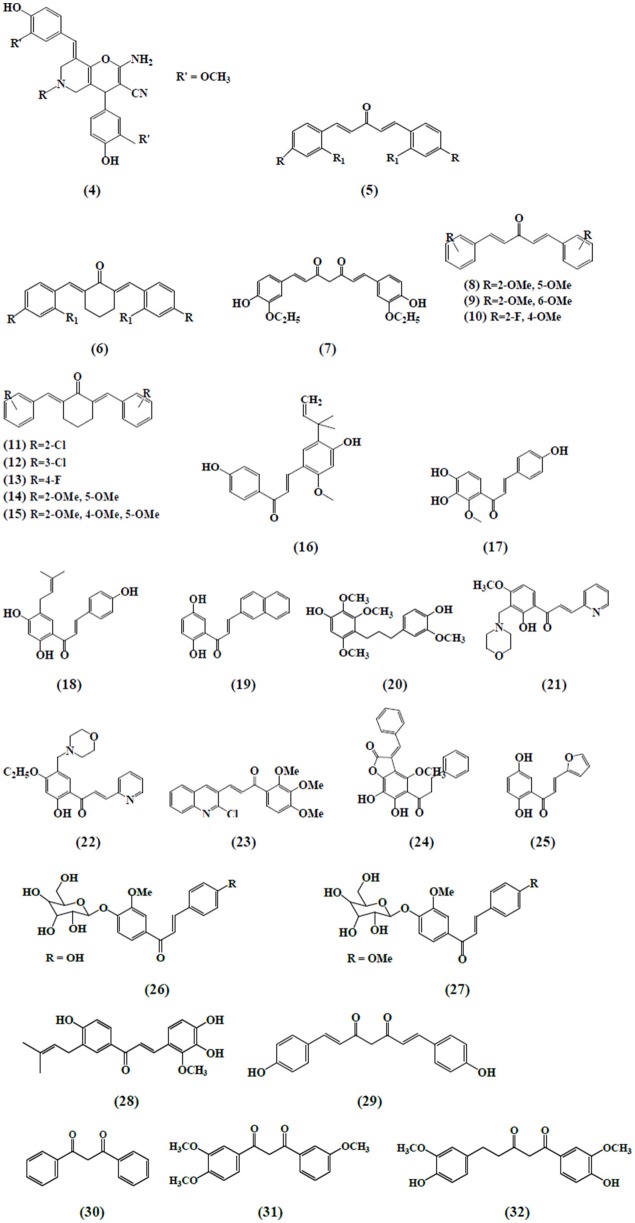
**Chemical structures of α,β-unsaturated carbonyl-based compounds with strong immunosuppressive effects on granulocytes**.

**Figure 4:**
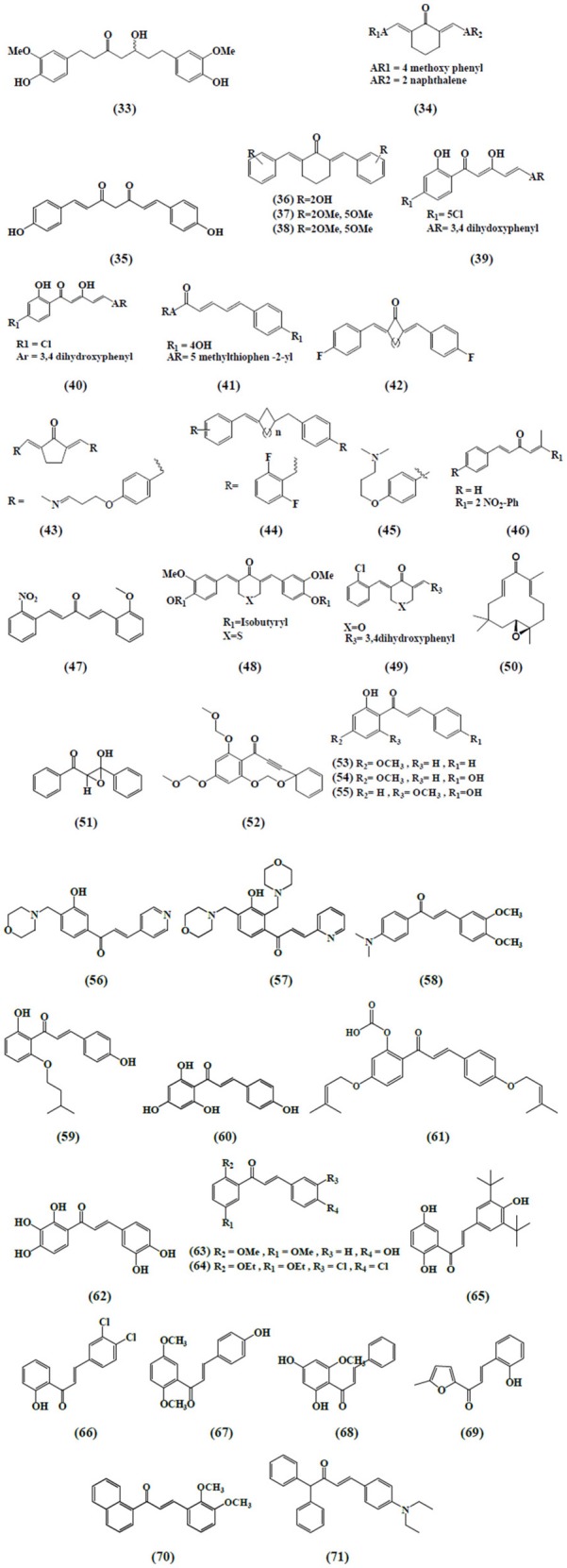
**Chemical structures of α,β-unsaturated carbonyl-based compounds with strong immunosuppressive effects on monocytes and macrophages**.

**Figure 5:**
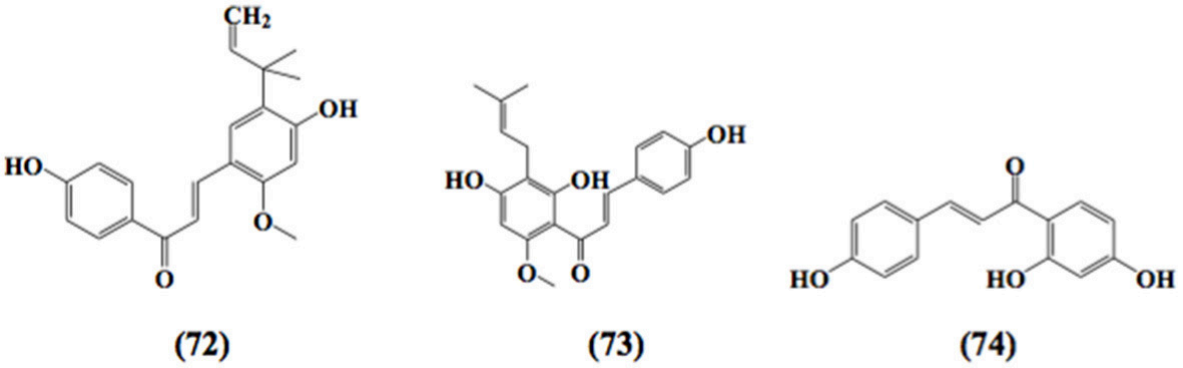
**Chemical structures of α,β-unsaturated carbonyl-based compounds with strong immunosuppressive effects on dendritic cells**.

Curcumin and its analogs have been shown to be able to arrest lymphocytes and antigen presenting cells (APC) in autoimmune diseases (Bright, 2007). As evident in arthritis, they seemed to inhibit neutrophil activation and subsequent reactive oxygen species (ROS) production along with suppression of nuclear factor kappa B (NF-κB) and other inflammatory cytokines released by white blood cells (Jackson et al., 2006; Jančinová et al., 2009). In a similar manner, curcumin exerted antioxidant effect, reducing hyperglycemia and also inhibited macrophages-mediated tumor necrosis factor (TNF) and interleukin-6 (IL-6) release, activated by dying or stressed B cells (Raine, 1990; Maradana et al., 2013). Moreover, it was identified that the increased number of CD8+ T cells has been reduced by the use of curcumin (Aggarwal and Harikumar, 2009). Correspondingly, chalcone and its derivatives have been recognized as exhibiting therapeutic potential in immune diseases by targeting NF-κB signaling pathway, kinase activities and others assumed to be involved in cancer, diabetes, inflammatory diseases, and certain infections (Zhou and Xing, 2015). Zerumbone found to possess significant effects on immune system through controlling the regulation of cytokines and chemokines secretion in immune cells (Keong et al., 2010; Shieh et al., 2015). Table 1 summarizes the effects of α,β unsaturated carbonyl-based compounds on immune cells. The chemical structures of natural α,β-unsaturated carbonyl-based compounds, and their analogs and derivatives which exhibited strong immunomodulatory effects on granulocytes (compounds 4–32), monocytes, and macrophages (compounds 33–71), and dendritic cells (compounds 72–74) are shown in Figures 3–**5**, respectively.

**Table 1:** **Effects of α,β unsaturated carbonyl-based compounds on immune cells**.

**Type of cells**	**α,β-unsaturated carbonyl based compounds**	**Model used**	**Key effects**	**References**
**CURCUMIN AND CURCUMIN ANALOGS**				
Neutrophils	Curcumin	Monkey	↓Production of ROS	Srivastava, 1989
	Curcumin	Murine	↓Raised level of PKC enzymes	Jančinová et al., 2009
	Curcumin	Chondrocyte	↓Inflammatory process	Jackson et al., 2006
	Curcumin	Human	↓Chemotaxis ↓IL 8 induced signal transduction pathway	Takahashi et al., 2007
	Curcumin	Human	↓Cytokine induced neutrophil chemo attractant	Lian et al., 2006
	Diarylpentanoid analogs	Human	↓ROS production	Bukhari et al., 2015
	Pyranopyridine,pyrazole pyridines and pyridopyrimidines	Human	↓ROS production	Al-Omar et al., 2005
	Synthetic curcumin analog	Human	↓ROS production	Youssef et al., 2004
Eosinophils	Curcumin	Murine	↓Migration and IL-4 and IL-5 synthesis	Moon et al., 2008
Mast cells	Curcumin	Murine	↓TNF-αand IL-4 ↓Histamine release	Lee et al., 2008; Kurup and Barrios, 2008
Monocytes /macrophages	Curcumin	Human	↓Adhesion to human endothelial cells	Kumar et al., 1998
	Curcumin	Human	↓TNF-α, IL-8, MiP-1α, MCP-1, IL-1β	Abe et al., 1999
	Curcumin	U937 cells	↓MCP-I	Lim and Kwon, 2010
	Curcumin	Murine	↓NO, TNF-α, IL-8	Bisht et al., 2009
	Curcumin	Murine	↓TLR4 mRNA expression	Matsuguchi et al., 2000
	Curcumin	U937 cells	↓Oxidative stress, IL-8, TNFα, IL-6	Jain et al., 2009
	Curcumin		↓Amyloid β-deposition	Hamaguchi et al., 2010
	Curcumin	THP-1	↓TNF-α,IL-1β,MCP-1,IL-8,MIP-1β, CCR5	Giri et al., 2004
	Curcumin	THP-1	↓Th1 cytokine response, ↓NO production	Bhaumik et al., 2000
	Curcumin	Human	↓Release of TNF and IL-1	Chan, 1995
	Dibenzoyl methane, Tetrahydrocurcumin and Trimethoxydibenzoylmethane	Murine	↓COX-2 expression, ↓5-LOX activity ↓PLA_2_ phosphorylation	Hong et al., 2004
	Bis dimethoxy curcumin	Murine	↓NO production	Kim et al., 2010
	Diacetyl curcumin manganese complex		↓NO production	Sumanont et al., 2004
	Hexahydrocurcumin	Murine	↓PGE_2_ synthesis, ↓iNOS	Lee et al., 2005
	Unsymmetrical monocarbonyl curcumin analogs	Human and Murine	↓PGE_2_ synthesis	Aluwi et al., 2016
	Demethoxycurcumin	Murine	↓NO production, ↓iNOS	Pae et al., 2008
	Diarylpentanoid analogs of curcumin	Murine	↓NO production	Lee et al., 2009
	Diarylpentene dione curcumin series	Murine	↓NO production	Leong et al., 2014
	2 benzoyl-6-benzylidene cyclohexanone curcumin series	Murine	↓NO production	Leong et al., 2015
	1,5 diphenylpenta-2,4-dien-1-one	Murine	↓TNF-α and IL-6	Liang et al., 2009a
	Monocarbonyl curcumin analogs	Murine	↓TNF, IL-1, IL-6,MCP-1,COX-2,PGE2,iNOS	Liang et al., 2009b
	Asymmetric monocarbonyl analogs	Murine	↓TNF- α, IL-6	Zhao et al., 2015
	Curcumin analogs	Murine	↓TNF- α, IL-6	Zhang et al., 2015
	Symmetric and asymmetric monocarbonyl curcumin analogs	Murine	↓TNF- α, IL-6	Zhang et al., 2014
Dendritic cells	Curcumin	Human	↓Proliferation of helper T-cells	Shirley et al., 2008
	Curcumin	Murine	↓Activities of regulating T-cells, ↓Oversuppression of CD 86, CD80, MHC II	Kim et al., 2005
	Curcumin	Murine	↓Of indoleamine 2,3 dioxygenase	Platt et al., 2010
	Curcumin	Murine	↓IDO production and T-cell proliferation	Jeong et al., 2009
	Curcumin	Murine	↓IL-12/23p40	Larmonier et al., 2008
T cells	Curcumin	Murine	↓Release of IL-2 and IFN-γ	Gao et al., 2004
	Curcumin	Jurkat	↓Proliferation of rat thymocytes	Sikora et al., 1997
	Curcumin	Human	↓Quiescent T-cells	Magalska et al., 2006
	Curcumin	Murine	↓OKT_3_ monoclonal antibody	Sikora et al., 2006
	Curcumin	Human	↓Proliferation	Deters et al., 2008
	Curcumin	Murine	↓Proliferation, ↓IL-2 and IFN-g	Gao et al., 2004
	Curcumin	Murine	↓IL-17 level,IL-17 mRNA expression	Xie et al., 2009
	Curcumin	Murine	↓TLR4 and TLR9 receptor expression	Chearwae and Bright, 2008
	Curcumin	Murine	↓IL-12 production	Natarajan and Bright, 2002
	Curcumin	Human	↓Lymphocyte proliferation	Moon et al., 2010
B cells	Curcumin		↓Proliferation and induce apoptosis	Han et al., 1999
	Curcumin	Murine	↓Proliferation	Decoté-Ricardo et al., 2009
	Curcumin	Murine	↓Proliferation and release of IgG_1_ and IgG_2_	Sharma et al., 2007
	Curcumin	Murine	↓Release of IgE antibodies	Kuramoto et al., 1996
	Curcumin	Human	↓B-cell immortalization	Ranjan et al., 1998
	Curcumin	Human	↑Apoptosis	Ranjan et al., 1999
Natural killer cells	Curcumin	Murine	↓LAK cell production	Gao et al., 2004
	Curcumin	Human	↓Release of IFN-γ and granzyme B	Bill et al., 2009
	Curcumin	TS/A cell line	↓Function and activity	Zhang et al., 2007
	Curcumin	Murine	↓Proliferation	Yadav et al., 2005
	Curcumin		↓Proliferation	Kim et al., 2005
**ZERUMBONE AND ITS DERIVATIVES**				
Neutrophils	Zerumbone	Murine	Blocking infiltration of PMNs	Chen et al., 2011
Eosinophils	Zerumbone	Murine	Prevent eosinophilic pulmonary infiltration	Shieh et al., 2015
Monocytes /macrophages	Zerumbone	Murine	↓COX-2 expression	Murakami et al., 2005
	Zerumboneoxide	Murine	↓NO production	Jang et al., 2005
	Zerumbone	Murine	↓NO production	Syahida et al., 2006
	Zerumbone	THP1 cells	↓AP-1 and NF-κB	Eguchi et al., 2007
	Zerumbone	Murine	↓PGE2 and NO production	Chien et al., 2008
	Zerumbone	Mouse monocytes and human breast tumor cells	↓RANKL-induced NF-κB activation, ↓IκBα kinase	Sung et al., 2009
	Zerumbone	Murine	↓COX-2 and iNOS	Ohnishi et al., 2009
	zerumbone and zerumbone 2,3-epoxide	Murine	↓NF-κB activation ↓NO production	Giang et al., 2009
	Zerumbone	U937 cells	U937 macrophages protection from TCDD and DDT mediated the toxic actions	Sciullo et al., 2010
	Zerumbone	Murine	↓mRNA, iNOS and IL-1β.	Igarashi et al., 2016
Dendritic cells	Zerumbone	Murine	↓Antigen presenting cells (type A)	Ganabadi and Kadir, 2009
	Zerumbone	Murine	↓Production of eotaxin, KC, IL-4, IL-5, IL-10, and IL-13	Shieh et al., 2015
T cells	Zerumbone		↑Apoptosis	Abdelwahab et al., 2011
	Zerumbone	Jurkat	↓T- cells proliferation ↑apoptosis	Rahman et al., 2014
**CHALCONE AND CHALCONE DERIVATIVES**				
Neutrophils	2-methoxy-4,4′ dihydroxy-5- α, α-dimethyl ally chalcone	Human	Biosynthesis of LTB4 and LTC 4	Kimura et al., 1988
	2-methoxy-3,4,4′ trihydroxychalcone	Human	Biosynthesis of LTB4 and LTC 4	Kimura et al., 1988
	Viscolin	Human	↓Free radical production and elastase secretion	Hwang et al., 2006
	Brousso chalcone A	Human	↓Respiratory burst	Wang et al., 1997
	2′,5′ dihydroxy-2-naphthyl chalcone		Neutrophil degranulation	Hsieh et al., 1998
	(E) -1-[2-hydroxy-4-methoxy-3-(morpholine-methyl)phenyl]-3-(pyridine-2-yl)prop-zen-1-one	Human	↓Production of superoxide ion and elastase release	Reddy et al., 2011
	(E) -1-[4-ethoxy-2-hydroxy-5-(-(morpholine-methyl)phenyl]-3-(pyridine-2-yl)prop-zen-1-one	Human	↓Production of superoxide ion and elastase release	Reddy et al., 2011
	Phenyl sulfonyl uranyl	Murine	↓Chemotaxis, ↓MPO, ↓ROS, ↓Elastase, ↓LTB4 production, ↓elastase, superoxide anion and LTB4	Araico et al., 2006
	1-(2,3,4 trimethoxyphenyl)-3-(3-2-chloroquinolinyl)-2-propen-1-one	Human and Murine	↓Elastase, superoxide anion and LTB4	De Leon et al., 2003
	Bratelactone	Human	↓Elastase release and superoxide synthesis	Wu et al., 2013
	2′,5′ dihydroxy-2-furfuryl chalcone	Murine	↓Respiratory burst	Wang et al., 2003
Basophils	Chalcone	Human	↓Histamine release	Middleton and Drzewiecki, 1984
	Liccochalcone	RBL-2H3 cells	↓Activation of ERK pathway	Tanifuji et al., 2010
	4′-o-B-D-glucopyronosyl-4-hydroxy-3′-methoxy chalcone	Human	↓Release of intra granular mediator	Middleton and Drzewiecki, 1984
	4′-o-B-D-glucopyronosyl-4-hydroxy-3′-dimethoxy chalcone	Human	↓Release of intra granular mediator	Middleton and Drzewiecki, 1984
Monocytes /macrophages	2′,4′,6′tris(methoxy methoxy)chalcone	Murine	↓NO production, ↓iNOS	Lee et al., 2006
	2,4,6 trimethoxy acetophenone	Murine	↓NO production	Chiaradia et al., 2008
	Trans -1,3-diphenyl-2,3-epoxypropane-1-one	Murine	↓NO and PGE2 production	Kim et al., 2013
	3-phenyl-1-(2,4,6-tris(dimethoxy)phenyl)prop-2-yn-1-one	Murine	↓AP-1 and ↓NO, ↓TNF- α	Park et al., 2009
	4-dimethylamino-3′,4′ dimethoxy chalcone	Mice	↓iNOS expression	Herencia et al., 2001
	2′,4-dihydroxy-6′-isopentlyoxy chalcone	Murine	↓TLP4 mediated NFK-β activation	Roh et al., 2011
	Sofalcone	Murine	↓TNF-α, MCP-1, NO	Tanaka et al., 2009
	Naringenin	Murine	↓TNF-α, MCP-1, NO	Hirai et al., 2007
	Okanin	Murine	↓iNOS and NO	Kil et al., 2011
	Phenyl Sulfonyl Uranyl chalcone	Murine	↓PGE2	Araico et al., 2006
Dendritic Cells	Licochalcone A	Human	↓Release of IL-6 and TNF-α	Kolbe et al., 2006
	Xanthohumol	Murine	↑Apoptosis	Xuan et al., 2010
T cells	Chalcone	Murine	↓Production and function of CD8^+^ cells	Schwartz and Middleton, 1984
	Xanthohumol	Murine	↓Proliferation of CD8^+^ T-cells and ↓Release of IL-2,IFN-γ and TNF-α	Gao et al., 2009
Dendritic cells	Zerumbone	Murine	↓Antigen presenting cells (type A)	Ganabadi and Kadir, 2009
	Licochalcone A	Human	↓Proliferation and release of IFN-γ	Barfod et al., 2002
	Naringenin	Murine	↓Release of Th-2 cytokines	Iwamura et al., 2010
	Naringenin	Murine	↑T-cell apoptosis, ↓mRNA expression of IL-2, IFN-γ and TNF-α	Fang et al., 2010

^*^↑, *Enhanced/Upregulation;* ↓, *Decreased/Inhibited/Downregulation*.

### Immunosuppressive effects of curcumin on granulocytes

Numerous lines of evidence suggest that curcumin may abate the neutrophil mediated inflammatory responses by varying different aspects of neutrophil function. Curcumin treatment inhibited zymosan and n-formyl-methionine-leucyl-phenylalanine (fMLP)-activated monkey neutrophils. It also suppressed the production of oxygen free radicals induced by arachidonic acid (Srivastava, 1989). The reduced production of oxygen free radicals by curcumin treatment may play an important role in rheumatoid arthritis. Jančinová et al. (2009) reported that curcumin inhibited the raised level of protein kinase C (PKC) isozymes by neutrophils in adjuvant induced arthritis. In the same way, curcumin inhibited the inflammatory processes initiated by crystal-induced neutrophil activation in a rheumatoid arthritis model (Jackson et al., 2006). The inhibition of transmigration and recruitment of neutrophils from blood vessels to the underlying liver tissue by curcumin has also been investigated in septic shock. The authors correlated the reduction of neutrophil's infiltration to the blocked expression of intercellular adhesion molecule 1(ICAM-1) and vascular cell adhesion protein 1 (VCAM-1) in the liver tissue with curcumin treatment (Madan and Ghosh, 2003). In another study, curcumin mediated inhibition of neutrophil's chemotaxis in a dose-dependent manner. It was reported that curcumin successfully impeded IL-8 (strong neutrophil chemoattractant) produced by monocytes and macrophage and also blocked IL-8 induced signal transduction pathway in neutrophils (Takahashi et al., 2007). Curcumin also reduced the level of cytokine-induced neutrophil chemoattractant (CINC) in acute lung injury. Based on the results, it was evidenced that curcumin exhibited anti-inflammatory activity by dampening CINC protein levels, which is similar in properties to human cytokine IL-8 and by diminishing the myeloperoxidase activity, a marker for damage and deterioration in lung tissues (Lian et al., 2006).

A study on the effect of curcumin on eosinophils in a murine model of asthma has shown that it inhibited the ovalbumin-(OVA) induced airway inflammation by three different mechanisms i.e., by suppressing the expression of inducible nitric oxide synthase (iNOS) and NO (nitric oxide) production responsible for significant lung tissue detriment; by hampering the recruitment of eosinophils and inflammatory cells that leads to the inflammation of airways; and by repressing the levels of IL-4 and IL-5 in broncho alveolar lavage fluid (Moon et al., 2008). In a model of cutaneous anaphylaxis, the antiallergic activity of curcumin was determined *in vivo*. The authors concluded that curcumin not only blocked the antigen-mediated activation of mast cells but also inhibited the secretion of cytokines like TNF-α and IL-4 by the mast cells, the underlying pathophysiology of atopic allergic reactions (Lee et al., 2008). In addition, another study indicated that curcumin inhibited the release of histamine from the mast cells, exhibiting anti-allergic effects in a murine model of allergy (Kurup and Barrios, 2008). During myocardial ischemia/reperfusion injury, it was shown that curcumin significantly lessen the myeloperoxidase activity, an index of neutrophils infiltration, hence alleviating cardiomyocytic apoptosis and global ischemia (Yeh et al., 2005).

A new series of curcumin analogs with α,β-unsaturated moiety i.e., pyrano pyridines, pyrazole pyridines, and pyrido pyrimidines has also revealed the antioxidant potential against free radical scavenging activity in isolated peripheral multinuclear neutrophils cells (PMNs) from the blood of healthy donors. Among the compounds tested, compound **4** exhibited the highest antioxidant activity (Al-Omar et al., 2005). Bukhari et al. (2015) assessed the inhibitory abilities of 17 diarylpentanoid analogs of curcumin against ROS production. The chemiluminescence assay was performed by using isolated PMNs and it was reported that compounds **5** and **6** showed strong inhibition on intracellular and extracellular ROS production by neutrophils with IC_50_ values ranging from 4.2 to 6.2 uM. In another study, a synthetic curcumin analog **(7)** exhibited a strong antioxidant activity by inhibiting neutrophils-mediated free radical scavenging activity against diphenylpicrylhydrazyl free radical test (DPPH; Youssef et al., 2004). Investigation on the immunomodulatory effects of 43 curcumin diarylpentanoid analogs revealed that compounds **8, 10, 13**, and **14** were found to possess marked inhibitory effect on the oxidative burst of PMNs as well as compounds **9, 10, 11, 12**, and **15** which exhibited potent inhibition of migration of PMNs (Jantan et al., 2012).

### Immunosuppressive effects of chalcone and its derivatives on granulocytes

Chalcone derivatives have also been found to play an important role on neutrophil's activities and functions. Kimura et al. (1988) reported that two chalcones i.e., 2-methoxy-4,4′-dihyroxy-5-α,α-dimethyl-allyl chalcone **(16)** and 2-methoxy-3,4,4′-trihydroxychalcone **(17)** isolated from *Glycyrrhiza glabra* inhibited the biosynthesis of leukotrienes, LTB4 and LTC4 in human neutrophils. The synthesis of leukotrienes was stimulated by calcium-ionophore and was considered to be involved in the pathology of various diseases. Broussochalcone A **(18)**, found in *Broussonetia papyrifera*, has showed a dose-dependent inhibition of fMLP and PMA elicited respiratory burst in activated rat neutrophils. Inhibition of PKC activity and suppression of NADPH oxidase activity were among the different contributing factors exhibiting the aforementioned effects (Wang et al., 1997).

Hsieh et al. (1998) reported the anti-inflammatory effects of a newly synthesized 2′,5′-dihydroxy-2-naphthylchalcone **(19)** which blocked fMPL-activated rat neutrophils which further blocked the release of β-glucuronidase and lysozyme. Human neutrophils mediated anti-inflammatory responses by viscolin **(20)**, a chalcone isolated from *Viscum coloratum* has been reported. It was observed that viscolin suppressed the free radicals production and elastase release by the fMLP stimulated human neutrophils and these effects were found to be associated with an enhanced levels of cellular cyclic adenosine monophosphate (cAMP) through the inhibition of cAMP-specific phosphodiesterase (PDE) degradative enzymes (Hwang et al., 2006). In another study, Mannich bases of heterocyclic chalcones i.e., (E)-1-[2-hydroxy-4-methoxy-3-(morpholinomethyl)phenyl]-3-(pyridin-2-yl)prop-2-en-1-one **(21)** and (E)-1-[4-Ethoxy-2-hydroxy-5-(morpholinomethyl)phenyl]-3-(pyridin-2-yl)prop-2-en-1-one **(22)** exhibited similar inhibitory effects (production of superoxide anion and elastases) in human neutrophils (Reddy et al., 2011).

Another chalcone derivative, phenylsulfonyl uranyl has been reported to reduce the chemotaxis process during inflammatory conditions. Also it seemed to be suppressing myeloperoxidase generation along with the production of elastases and oxygen free radicals in neutrophils stimulated by different inducers including PMA, fMLP, and cytochalasin B. These effects are correlated with the inhibition of LTB4, an important leukotriene synthesized by the enzyme 5 lipoxygenase during the lipoxygenase pathway (Araico et al., 2006). Furthermore, De Leon et al. (2003) also studied the aforementioned parameters with the synthetic chalcone derivative, 1-(2,3,4-trimethoxyphenyl)-3-(3-(2-chloroquinolinyl)-2-propen-1-one **(23)** and documented its inhibitory function on neutrophils. The anti-inflammatory effects through the inhibition of CD11b expression, elastase release and free radicals generation in fMLP activated neutrophils have been exhibited by a novel chalcone, bratelactone, **(24)** isolated from *Fissistigma bracteolatum*. The observed underlying mechanism involved the blockage of calcium signaling, particularly store operated calcium entry block (SOCE) which is considered of paramount importance in activation of human neutrophils (Wu et al., 2013). Phospholipid D, an important component involved in the synthesis of phosphatidic acid, required for the activation of NADPH oxidase which is further important for neutrophil activation. Therefore, the inhibition of phospholipase D activation, by blocking PKC, Rho A, and ADP-ribosylation factor (ARF) membrane association is one of the critical mechanism in suppressing neutrophil's function. Another chalcone derivative, namely, 2′, 5′-dihydroxy-2-furfurylchalcone (DHFC) **(25)** has manifested dose-dependent inhibition of oxidative burst in fMLP activated neutrophils employing same mechanism as stated earlier (Wang et al., 2003).

Middleton and Drzewiecki (1984) revealed the inhibitory effects of chalcones on the release of histamine, an important cell derived inflammatory mediator, by human basophil. The authors studied the effect of different inducers antigen, anti-IgE, concanavalin (CON-A), ionophore, fMLP, and tetrade-canoylphorbol acetate to stimulate basophil cells. Likewise, Itoh et al. (2010) reported the anti-allergic role of two chalcone derivatives i.e., 4′-O-β-D-glucopyranosyl-4-hydroxy-3′-methoxychalcone **(26)** and 4′-O-β-D-glucopyranosyl-3′,4′-dimethoxychalcone (**27**) isolated from *Brassica rapa* L. using rat RBL-2H3 basophilic cells. The results revealed that chalcones inhibited the release of intra granular mediators in response to an antigen stimulus. Another study demonstrated the same inhibitory effects in rat RBL-2H3 basophilic cells by a chalcone derivative namely; licochalcone D **(28)** isolated from the root *Xinjiang liquorice*. These antiallergic effects accounted for their capability to inhibit the activation of the extracellular signal regulated kinase pathway (ERK) and extracellular Ca^2+^ influx (Tanifuji et al., 2010).

### Immunosuppressive effects of zerumbone on granulocytes

Chen et al. (2011) demonstrated the effect of zerumbone on ultraviolet B (UVB)-induced corneal damages in female imprinting control region (ICR) mice where infiltration of PMNs was significantly blocked by the treatment of dietary zerumbone. The UVB-induced corneas were found to contain infiltrative PMN leukocytes in stroma and aqueous humor. Few PMN leukocytes were shown to attach with endothelial layer possessing a significant risk of attack to endothelial cells of cornea. Zerumbone was found to ameliorate UVB-treated corneal damages. It has been evidenced that infiltration of PMN leukocytes was suppressed with dietary zerumbone in a concentration-dependent manner. Recently, Shieh et al. (2015) reported that zerumbone has the ability to prevent eosinophilic pulmonary infiltration. Treatment with zerumbone inhibited the airway inflammation followed by reducing eotaxin production in murine model of asthma. Eotaxin is a most significant chemokine for recruiting eosinophils. Investigating this chemokine level in bronchoalveolar lavage fluid (BALF) revealed that treatment with zerumbone remarkably inhibited the eotaxin secretion. This inhibition of eotaxin level thought to be due to the direct or indirect influence exhibited on recruiting eosinophils. Furthermore, the less infiltration and activation of eosinophils in the lung of zerumbone-induced mice thought to have revealed in inhibited IL-5 expression in BALF as eosinophils are also considered for the source of IL-5.

### Immunosuppressive effects of curcumin on monocytes and macrophages

The inflammatory responses mediated by monocytes and macrophages play a pivotal role in chronic inflammatory conditions. Kumar et al. (1998) reported that curcumin exhibited anti-inflammatory effects by blocking the adhesion of monocytes on human endothelial cells. These effects have been attributed to the inhibition of NF-κB which is responsible for the expression of proteins like ICAM-1, VCAM-1, and endothelial cell leukocyte adhesion molecule-1 (ELAM-1) on monocytes, induced by TNF. Also it has shown concentration-dependent inhibition of certain inflammatory cytokines secretion i.e., TNF-α, IL-8, macrophage inflammatory protein 1 alpha (MIP-1α), monocyte chemoattractant protein (MCP-1) and IL-1β after monocytes and macrophages had been stimulated by PMA or lipopolysaccharide (LPS; Abe et al., 1999). Lim and Kwon (2010) also revealed similar findings in their study that curcumin suppressed the expression of MCP-1 in PMA-induced U937.

Monocytic cell lineage. MCP-1 is a key factor in macrophages migration toward the site of infection and this inhibition has been subjected to the blocking of ERK /and NF-κB transcriptional activity. Another study was conducted to determine the anti-inflammatory role of curcumin by assessing different functions of macrophages *viz* NO production, phagocytosis and cytokines release. It was recognized that this multi targeting agent inhibited the synthesis of NO along with the blocking of TNF-α and IL-8 secretion by raw macrophages triggered by LPS. On the other hand, it enhanced the phagocytic activity and CD14 surface expression which was suggested as an indirect role of curcumin with unknown mechanism (Bisht et al., 2009).

Matsuguchi et al. (2000) observed the effects of curcumin on expression of toll like receptors in mouse macrophages in response to intraperitoneal injection of LPS. The results showed that pretreatment with curcumin significantly suppressed TLR4 mRNA expression which was subjected to NF-κB inhibition, suggesting that NF-κB is essential for this process. In diabetic condition, a massive increase in the inflammatory cytokines has been reported. In a model of streptozotocin-induced diabetic rats, the anti-inflammatory effect of curcumin was evaluated on the secretion of various inflammatory cytokines and also these effects were studied by using human promonocytic U937 cell line. Treatment with curcumin not only reduced enhanced levels of IL-8, TNF-α, IL-6, MCP-1 secreted as a result of increased glucose levels but also reduced cellular oxidative stress (Jain et al., 2009). Alzheimer's disease is a disorder presented mainly with massive amyloid-β (Aβ) peptide and monocytes and macrophages accumulation in the affected tissue. Increased levels of cytokines, TNF-α, and IL-1β and chemokines MCP-1, IL-8, and MIP-1β have also been observed. The ability of curcumin to modulate β-secretase and AChE activities led to decreased amyloid-β deposition and macrophages accumulation (Hamaguchi et al., 2010). Correspondingly, Giri et al., 2004) also explained the anti-inflammatory role of curcumin in Alzheimer's disease. It was reported that curcumin inhibited amyloid-β peptide aggregation and Aβ-induced expression of TNF-α, IL-1β, MCP-1, IL-8, MIP-1β, and CCR5 in THP-1 monocytic cells. In a model of subcutaneously transplanted AK-5 tumors, curcumin led to the suppression of type 1 T helper (Th1) cells cytokine response and NO production by macrophages, suggesting that the host macrophages played an important modulatory role in the remission of AK-5 tumor (Bhaumik et al., 2000). Curcumin has also been found to hamper the release of TNF and IL-l as a result of inhibition of NF-κB by human monocytic macrophages triggered by LPS. This pleiotropic cytokines play a crucial role in different acute and chronic inflammatory diseases e.g., atherosclerosis, AIDS and autoimmune disorders (Chan, 1995). Likewise, Gao et al. (2004) also investigated the suppression of aforementioned cytokines in peritoneal macrophages by diferuloylmethane.

### Immunosuppressive effects of curcumin analogs and derivatives on monocytes and macrophages

The reduced level of heme oxygenase-1 expression in RAW264.7 cells treated with bisdemethoxycurcumin **(29)** was found as one of the mechanisms of LPS-mediated NO production inhibition. Also this anti-inflammatory mechanism involved Ca^2+^/calmodulin- CaMKII-ERK1/2-Nrf2 cascade in RAW264.7 macrophages (Kim et al., 2010). Anti-inflammatory activity mediated by manganese complexes of curcumin (CpCpx) and diacetylcurcumin manganese complex (AcylCpCpx) exhibited enhanced NO radical scavenging activity as compared to their parent compounds, curcumin and acetylcurcumin, respectively (Sumanont et al., 2004). Skin wound healing involves androgen receptor signaling and these receptors have been found in different cell types including keratinocytes, dermal fibroblasts, and infiltrating macrophages. Lai et al. (2009) evaluated the curcumin derivative ASC-J9, against general and myeloid-specific AR knock out (ARKO) mice. The study concluded that the topical application of ASC-J9 cream in mice caused accelerated wound healing by inhibiting the androgen receptor activity and suppressed expression of local TNF-α expression. Anti-inflammatory activities mediated by naturally occurring curcumin and β-diketone derivatives namely dibenzoylmethane **(30)**, triethoxydibenzoylmethane **(31)**, tetrahydrocurcumin **(32)** using RAW 264.7 macrophages cell line have been investigated. The observed parameters included the release of AA and its metabolites (Hong et al., 2004). It was concluded from the results that curcumin and its analogs significantly affected the AA metabolism by blocking cytosolic phospholipase A2 (cPLA2) phosphorylation, suppressing cyclooxygenase 2 (COX-2) expression and decreasing the catalytic activities of 5 lipoxygenase (LOX) in LPS-stimulated raw macrophages.

Lee et al. (2005) evaluated newly synthesized α,β-unsaturated curcumin analogs i.e., diarylheptanoid analogs and diarylheptylamine analogs as COX-2 inhibitors. These compounds strongly inhibited COX-2 catalyzed biosynthesis in LPS-stimulated macrophages as a model system. Among all the compounds, hexahydrocurcumin **(33)** was the most potent one with an IC_50_ value of 0.7 μM. Also the authors reported that the above mentioned curcumin analogs could inhibit iNOS responses of LPS-stimulated macrophages exhibiting anti-inflammatory activities. A series of unsymmetrical monocarbonyl curcumin analogs were synthesized and their anti-inflammatory effects were determined. Human and murine macrophages stimulated by LPS to determine the effects on prostaglandin E2 (PGE2) production were used. The results suggested that compound **34** exhibited strong inhibition on the production of PGE2 in both cell lines (Aluwi et al., 2016). Another study investigated a more stable synthetic curcumin analog, dimethoxycurcumin (**35)**, for NO production assay and compared with curcumin and bisdemethoxycurcumin (**29**). LPS activated macrophages were used to evaluate the inhibition of NO production and iNOS expression. The results showed that compound **35**, curcumin and compound **29** inhibited NO production, iNOS expression with compound **35** being the most effective, followed by curcumin and compound **29**, confirming that α,β-unsaturated carbonyl group is an important reactive structure of curcumin analogs. Additionally, this compound also revealed the inhibition of NF-κB in LPS-activated raw macrophages. Further mechanistic elucidation demonstrated that diminished NF-κB activation was achieved by blocking IκBα phosphorylation and IKK-α activity (Pae et al., 2008).

A newly synthesized series of curcumin related diarylpentanoid analogs have been shown to possess anti-inflammatory activity. Among the 46 compounds tested, three curcumin-like diarylpentanoid analogs which were **36, 37**, and **38** showed remarkably high inhibitory effect upon NO production in RAW 264.7 cell line, as compared to positive control and curcumin. Inhibition of target protein was suggested as the mechanism responsible for the anti-inflammatory property of these compounds (Lee et al., 2009). Likewise, a series of 97 diarylpentanoid derivatives with diarylpentenedione series (halogenated, methoxylated, and polyphenolic) were synthesized and screened through NO suppression assay for their anti-inflammatory activity using interferon gamma (IFN-γ)/(LPS)-stimulated macrophages cells. However, compound **39** demonstrated the most significant NO suppression activity. The authors concluded their study by reporting that the diarylpentanoid structure with preserved ethylene and β-diketone moieties was a primary direction and should be investigated further toward finding new anti-inflammatory agents (Leong et al., 2014).

In another study, compound **40** belonging to 2-benzoyl-6-benzylidenecyclohexanone curcumin analogs series was evaluated for NO inhibition in LPS/IFN-induced RAW 264.7 macrophages model and was found to exhibit the highest activity with an IC_50_ value of 4.2 ± 0.2 μM (Leong et al., 2015). Analogously, in a model of IFN-γ/LPS-activated RAW 264.7 cells, similar results have been seen when a series of 1, 5-diphenylpenta-2, 4-dien-1-one analogs was evaluated for anti-inflammatory activity by measuring their NO inhibition activity (Faudzi et al., 2015). The most promising NO-inhibitory activity was displayed by compound **41**, a 5-methylthiophenyl-bearing analog. As a sequence from previous study, the authors investigated the bioactivity of a newly synthesized compound **42** against LPS-induced TNF-α and IL-6 secretion by using mouse J774.1 macrophages (Liang et al., 2009a). Liang et al. (2009b) evaluated another 44 mono-carbonyl analogs for the inhibitory activities against LPS-induced TNF-α and IL-6 release in the macrophages. Among these compounds, three were selected to further evaluate the inhibitory effects on LPS-induced TNF-α, IL-1, IL-6, MCP-1, COX-2, PGES, iNOS, and p65 NF-κB mRNA production and it was observed that compound **43** exhibited significant inhibitory effects.

Newly synthesized 23 mono-carbonyl analogs of curcumin demonstrated anti-inflammatory activity in one study and among these, compounds **44** and **45** were recorded as the most potent analogs exhibiting anti-inflammatory activity in a dose-dependent manner. Their anti-inflammatory abilities were examined against TNF-α and IL-6 release in LPS-stimulated RAW 264.7 macrophages (Zhao et al., 2010). In another study, the author found that compound **46**, a 2-nitro-substituted analog significantly (with the highest inhibition rate) suppressed the production of TNF-α and IL-6 in LPS-stimulated macrophages. Further investigation into the possible mechanism revealed that the anti-inflammatory activity of the analog might be associated with its inhibition against LPS-induced NF-κB and ERK pathway activation (Zhao et al., 2015). A very similar result was reported by evaluating the newly designed curcumin analogs for their anti-inflammatory effects in mouse peritoneal macrophages using ELISA kits. Active compound **47** exhibited highest inhibition on LPS-induced production of TNF-α and IL-6 and showed high chemical stability confirmed by UV absorption spectra (Zhang et al., 2015). In another study, the author classified the newly synthesized 34 monocarbonyl curcumin analogs into two series, symmetric monocarbonyl analogs of curcumin (SMAC) and asymmetric monocarbonyl analogs of curcumin (ASMAC). Among the analogs, the symmetrical heterocyclic type (compound **48** and **49**) displayed the strongest inhibition against LPS-induced TNF-α and IL-6 release in mouse macrophages (Zhang et al., 2014).

### Immunosuppressive effects of zerumbone and its derivatives on monocytes and macrophages

Zerumbone exhibited immunosuppressive effects on LPS-induced COX-2 expression in RAW264.7 murine macrophages followed by several mode of action (Murakami et al., 2005). Interestingly, a study reported that zerumbone derivatives, 5-hydroxyzerumbone and zerumboneoxide **(50)** suppressed LPS-induced NO production in RAW 264.7 cells with IC_50_ values of 14.1 and 23.5 μM, respectively (Jang et al., 2005) (Figure 4). In IFN-γ/LPS induced RAW 264.7 macrophages model, zerumbone also displayed more than 90% inhibition of NO production at the concentration of 50 μM (Syahida et al., 2006). Another study was carried out with differentiated Caco-2 cell lines to investigate the inhibitory effects of 16 selected food items on tissue plasminogen activator (TPA)-induced LOX-1 mRNA expression in THP-1 monocyte-like human cells and it was reported that zerumbone suppressed the transcriptional activities of activator protein (AP-1) and NF-κB (Eguchi et al., 2007). Zerumbone has also been documented as an inhibitor of PGE2 and NO production (Chien et al., 2008). Sung et al. (2009) found zerumbone to inhibit receptor activator for NF-kB ligand (RANKL) induced NF-κB activation in the mouse monocytes through the suppression of activation of IκBα kinase, IκBα degradation and IκBα phosphorylation as well as found to suppress osteoclast formation induced by human breast tumor cell lines.

The inhibition of pro-inflammatory genes (COX-2 and iNOS) expression and enhanced detoxification genes gluthathione-S-transferases (GSTP1) and nicotinamide quinone oxidoreductase (NQO1) in RAW264.7 murine macrophages have been revealed by zerumbone (Ohnishi et al., 2009). In another comparative study, zerumbone and zerumbone 2,3-epoxide inhibited NF-κB activation and NO production in RAW 264.7 murine macrophages with respective IC_50_ values of 1.97 μM ± 0.18 and 30.11 μM ± 4.10 in activation of NF-κB assay and 3.58 μM ± 0.46 and 34.7 μM ± 3.72 in the NO production assay (Giang et al., 2009). Likewise, 5-hydroxyzerumbone found to suppress NO production in a dose-dependent manner with IC_50_ value of 19 μM (Min et al., 2009). In addition to RAW 264.7 macrophages, zerumbone has also been identified as a potential agent in protecting U937 macrophages from tetrachlorodibenzo-p-dioxin (TCDD) and dichlorodiphenyltrichloroethane (DDT)-mediated toxic actions (Sciullo et al., 2010). An intriguing study narrated the zerumbone-induced heat shock factor 1 (HSF-1) activation in stimulated macrophages for its anti-inflammatory functions. The outcomes revealed that zerumbone enhanced cellular protein aggregates and found to promote nuclear translocation of HSF-1 for heat shock protein (HSP) expression. Moreover, down-regulation of HSF1 attenuated the inhibitory effects of zerumbone on expressions of mRNA and proteins of pro-inflammatory genes, including iNOS and IL-1β (Igarashi et al., 2016).

### Immunosuppressive effects of chalcone and its derivatives on monocytes and macrophages

Inhibition of NO synthesis by altering the expression of induced enzymes is one of the essential mechanisms to attain anti-inflammatory activity. Synthetic chalcones with α,β-unsaturated moiety, namely 2′,4′,6′-tris(methoxymethoxy) chalcone has also been reported to retard NO production by inhibiting iNOS expression along with the inhibition of NF-κB translocation into the nucleus in RAW 264.7 macrophages fostered by LPS (Lee et al., 2006). In a similar manner, 14 synthetic chalcones derived from 2,4,6-trimethoxyacetophenone revealed inhibitory action, *in vitro*, in relation to NO production in murine macrophages of the line RAW 264.7 induced by bacterial LPS (Chiaradia et al., 2008). Analogously, trans-1,3-diphenyl-2,3-epoxypropane-1-one **(51)**, another chalcone derivative has disclosed the reduced production of NO and PGE2 in RAW 264.7 macrophages stimulated by LPS as a consequence of suppressed iNOS and COX-2 expression. Meanwhile, the reduced production of proinflammatory cytokines (TNF-α, IL-1β, and IL-6) has also been documented by compound 51 which was associated with the blocking of NF-κB activities as well as mitogen activated protein kinase (MAPK) phosphorylation. These two mechanisms seemed to be prominent in the pathophysiology of several inflammatory conditions (Kim et al., 2013).

The anti-inflammatory role of chalchone derivative i.e., 3-phenyl-1-(2,4,6-tris(methoxymethoxy)phenyl)prop-2-yn-1-one **(52)** was described by assessing the activity of AP-1 along with NO synthesis in murine RAW 264.7 macrophages (Park et al., 2009) The study revealed that the compound significantly suppressed the expression of certain AP-1 protein subunits induced by LPS and hence reduced the transcriptional activity of AP-1. The researchers also reported a dose-dependent abrogation of LPS-induced TNF-α secretion by a chalcone derivative (Franck et al., 2008). The inhibition of LPS stimulated NO and TNF-α production in RAW 264.7 macrophages by 2′-hydroxy-4′-methoxychalcone **(53)**, 2′,4-dihydroxy-4′-methoxychalcone **(54)**, and 2′,4-dihydroxy-6′-methoxychalcone **(55)** was attributed to the suppression of NF-κB and AP-1 activation that are considered indispensable for iNOS and cytokine gene expression (Figure 4; Ban et al., 2004).

Recently, a study also expressed that chalchone derivatives, (E)-1-[3-Hydroxy-4-(morpholinomethyl)phenyl]-3-(pyridin-4-yl)prop-2-en-1-one **(56)** and (E)-1-[3-Hydroxy-2,4-bis(morpholinomethyl)phenyl]-3-(pyridin-2-yl)prop-2-en-1-one **(57)** caused the prevention of NO synthesis in LPS- and IFN-γ-stimulated RAW 264.7 macrophages (Reddy et al., 2011). Besides, 4-dimethylamino-3′,4′-dimethoxychalcone **(58)** showed iNOS expression downregulation in cultured peritoneal macrophages extracted from mice (Herencia et al., 2001). On the other hand, a chalcone derivative, 2′,4-dihydroxy-6′-isopentyloxychalcone (JSH) **(59)** exhibited the inhibition of toll like receptors (TLR4)-mediated NF-κB activation in LPS-induced macrophages. These tolls like receptors (TLR-4) are usually manifested in macrophages and are imperative for eliciting innate immune responses (Roh et al., 2011). Tanaka et al. (2009) and Hirai et al. (2007) depicted that naringenin chalcone **(60)** and sofalcone (20-carboxymethoxy-4,40-bis(3-methyl-2-butenyloxy)chalcone) **(61)** showed their anti-inflammatory abilities by dose-dependent inhibition of LPS-induced TNF-α, MCP-1, and NO production in RAW 264.7 macrophages. The inhibitory effect of sofalcone on NO production was attributed to the activation of an enzyme (HO-1 expression) which plays a pivotal role in regulating the inflammatory condition (Tanaka et al., 2009).

Analogously, Kil et al. (2011) also reported, a naturally occurring chalchone, okanin **(62)**, isolated from the genus Bidens and 3-penten-2-on inhibited LPS-induced iNOS expression and NO production in RAW264.7 macrophages through upregulation of HO-1 expression. Phenylsulfonyl uranyl chalcone derivative has been found to inhibit LPS-induced PGE2 production in RAW 264.7 macrophages in one study. These inhibitory effects of chalcone derivatives on the synthesis of PGE2 were correlated to the inhibition of COX-2 function selectively (Araico et al., 2006). The two chalcone derivatives 2′,5′-dimethoxy-4-hydroxychalcone **(63)** and 3,4-dichloro-2′,5′-diethoxychalcone **(64)** and another three synthetic chalcones 3,5-di-tert-butyl-2′,4,5′-trihydroxychalcone **(65)**, 2′-hydroxy-3,4-dichlorochalcone **(66)**, and 2′,5′-dimethoxy-4-hydroxychalcone **(67)** illustrated the retardation of NO synthesis in RAW 264.7 macrophages and microglial cells activated by LPS (Ko et al., 2003; Won et al., 2005).

Cardamonin, a chalcone derivative isolated from *Artemisia absinthium* L., and chemically known as 2′,4′-dihydroxy-6′-methoxychalcone **(68)**, exerted anti-inflammatory effects in LPS -stimulated THP-1 monocytic cells/ IFN-γ activated RAW 264.7 macrophages. The inhibitory effects were seen on TNF-α production and iNOS protein expression which was subjected to restricted interaction of NF-κB to the DNA (Hatziieremia et al., 2006). 3-(2-hydroxyphenyl)-1-(5-methyl-furan-2- y-1)propenone (HMP) **(69)**, revealed suppression of p38/ATF-2 and AP-1 signaling pathways, another mechanism, responsible for LPS mediated dose dependent inhibition of NO generation in RAW 264.7 macrophages (Liew et al., 2011). Two synthetic chalchones i.e., 3-(2,3-dimethoxy-phenyl)-1-naphthalene-1-yl-propenone **(70)** 4-(4-diethylamino-phenyl)-1,1-diphenyl-but-3-en-2-one **(71)** and two β-chlorovinyl chalcones have been documented to be able to suppress IL-6 and TNF-α release in LPS triggered RAW 264.7 macrophages and THP-1 cells (Bandgar and Gawande, 2010; Jantan et al., 2014).

### Immunosuppressive effects of curcumin on dendritic cells

Dendritic cells (DCs) are of paramount importance in producing several immune responses and hence play fundamental role in different chronic pathological conditions. These cells have a pivotal role in initiating cell-mediated immunity and modification of dendritic cell's functions might be advantageous in eradicating immune disorders. A number of studies have investigated the immunomodulatory role of curcumin on dendritic cell's activities and functions. Curcumin owing to its immunosuppressive effects prevented the dendritic cells to respond to immune stimulants like LPS and proliferation of helper T cells. These inhibitory effects were subjected to suppression of maturation marker, cytokine and chemokine expression which further blocked the process of migration and endocytosis (Shirley et al., 2008). A study showed that curcumin could modulate dendritic cells mediated distinct immune responses in autoimmune disorders. It was reported that curcumin not only impeded the dendritic cell's maturation but also inhibited the subsequent stimulatory immune functions for regulating T cells activities. Also the over expression of CD86, CD80 and major histocompatibility complex II (MHCII) surface receptors were suppressed in LPS-stimulated dendritic cells after treatment with curcumin. It was observed that a dose of 50 μM proved to be lethal for DCs, reflecting the reactiveness of different immune cells to variable curcumin doses (Kim et al., 2005).

Indoleamine 2, 3-dioxygenase (IDO) is a heme containing immunomodulatory enzyme encoded by IDO1 gene and produced by several immunomodulatory cells like macrophages. On the other hand, IFN-γ, a cytokine, plays double role as an inducer and regulator of immune and inflammatory responses. It also regulates various responses of dendritic cells particularly, it shape up monocyte derived dendritic cells in cancer therapy and hence manage the polarizing activities of helper T cells 1. In one study, it was seen that IFN-γ enhanced IDO production in murine bone marrow derived DCs while curcumin displaying anti-inflammatory activity suppressed IDO production in above-mentioned cell line, further inhibiting IDO-mediated T cell proliferation. Modulation of janus kinase 1 (JAK) and PKC signaling pathway were recognized as mechanism for anti-inflammatory effects of curcumin in IFN-γ stimulated murine dendritic cells (Jeong et al., 2009).

In an *in vitro* model of the LPS-induced IL-12/23p40, difurolylmethane suppressed the production of IL-12/23p40 from the dendritic cells. The data from this study also suggested that curcumin depends on IL-10 to produce inhibitory immune responses and therefore by acting synergistically even at their suboptimal doses suppressed the LPS-induced IL-12/23p40 production from DCs (Larmonier et al., 2008). Curcumin has also been found to significantly inhibit release of LPS-stimulated inflammatory cytokines like IL-1B, IL-6, and TNF-α along with expression of IL-12 and dendritic cell's maturation which ultimately lead to poor cell -mediated immune responses and more likely to serve as a promising therapy in TH-1 mediated autoimmune disorders (Brouet and Ohshima, 1995).

### Immunosuppressive effects of zerumbone on dendritic cells

In a study, the role of zerumbone was investigated on the infiltration of major histocompatibility complex (MHC) class II cells in collagen-treated osteoarthritis of rats. The outcomes revealed that zerumbone could inhibit APC (type A) followed by decreasing inflammatory process in osteoarthritis (Ganabadi and Kadir, 2009). Recently, another study documented the immunomodulatory effects of zerumbone on APCs (DCs) *in vitro* and its therapeutic role against OVA-induced T helper 2 (Th2)-mediated asthma in mouse model. The research group reported that LPS-activated bone marrow-derived dendritic cells along with zerumbone could enhance the proliferation of T cells and polarization of Th1 cell. In an animal model, oral administration of zerumbone produced lower OVA-specific IgE (IgE) and increment of IgG2a antibody production. Furthermore, treatment with zerumbone was found to decrease the production of eotaxin, keratinocyte-derived chemokine (KC), IL-4, IL-5, IL-10, and IL-13, and increase Th1 cytokine i.e., IFN-γ production in asthmatic mouse model (Shieh et al., 2015).

### Immunosuppressive effects of chalcone and its derivatives on dendritic cells

Licochalcone A **(72)** blocked the secretion of IL-6 and TNF-α in LPS stimulated immature monocyte-derived human dendritic cells (Kolbe et al., 2006). In a detailed study, another chalcone, xanthohumol **(73)**, exerted apoptotic activity in mice dendritic cells. This pharmacological effect has been recognized as a potential target to alleviate inflammation, as xanthohumol caused the activation of caspase 8 and caspase 3 which in turn led to DNA frag-mentation and destruction of intracellular proteins and hence caused apoptosis in dendritic cells (Xuan et al., 2010).

## Immunosuppression of natural α,β-unsaturated carbonyl-based compounds on the adaptive immune system

### Immunosuppressive effects of curcumin on T cells

Several studies have also documented the role of α,β-unsaturated compounds on lymphocytes. Curcumin inhibited the expression of inflammatory cytokines like IL-2 and IFN-Υ by T lymphocytes isolated from mouse spleen cells. Basically, the inhibition of transcription factor i.e., NF-κB by curcumin was one of the bottom line mechanism in suppressed cytokine release ultimately concluding that this multi-targeting agent mediated most of the immunosuppressive effects by obstructing NF-κB target genes (Gao et al., 2004). Similarly, another study reported that curcumin abrogated the proliferation of rat thymocytes stimulated by mitogen Con A in addition to inhibitory effects of curcumin on Con A triggered Jurkat T cell line. These antiproliferative effects of curcumin have been attributed to arrest AP-1 transcription factor activation (Sikora et al., 1997). However, in disagreement to the above mentioned study, it was also reported that curcumin could also cause cell death not only in proliferating human lymphocytes but also in normal non-dividing quiescent T cells via activation of caspase-3 protein. Although, proliferating T cells were more prone to damage as compared to quiescent T cells (Magalska et al., 2006; Sikora et al., 2006). Moreover, in another study the proliferation of T and B cells activated by OKT3 monoclonal antibody (a human TCR/CD3 complex Ab) was significantly blocked by curcumin (Deters et al., 2008). Curcumin also showed the notable inhibition of human T cells when these cells were stimulated by different inducers like PMA, CD28, phytohaemagglutinin (PHA; Ranjan et al., 1998).

Curcumin owing to its immunosuppressive effects inhibited the proliferation of helper T cells by preventing the dendritic cells to respond to immune stimulants like LPS. These inhibitory effects were subjected to suppression of maturation marker, cytokine, and chemokine expression which further blocked the process of migration and endocytosis (Shirley et al., 2008). Another study highlighted the effect of different doses of curcumin on Con A-stimulated proliferation of splenic cells *in vitro*. It was observed that at 6.25 μM concentration curcumin exhibited a significant decrease in proliferation while a complete blockage of proliferation was observed at 12.5 μM in splenic T cells. In addition, curcumin was found to suppress the release of IL-2 and IFN-Υ by splenic T lymphocytes as a result of inhibition of NF-κB target genes rather than affecting the levels of constitutively expressed NF-κB (Gao et al., 2004). One study documented the multiple roles of curcumin using T cells activated by different stimuli. The IL-12-induced STAT4 phosphorylation was found to be reduced by curcumin while the same effect was enhanced in response to IFN-β-induction. On the other hand, curcumin lowered IL-12 induced IFN-γ production and IL-12 Rβ1 and β2 expression, whereas it increased IFN-α-induced IL-10 and IFNAR1 expression (Fahey et al., 2007).

Interleukin 17 is a pro-inflammatory cytokine produced by T cells which further triggers T cells and other immune cells to produce a diversity of cytokines, chemokines, and cell adhesion molecule and plays a key role in the progression of encephalomyelitis. In an experimental model of encephalomyelitis, oral treatment of curcumin reduced IL-17 levels along with IL-17 mRNA expression, production of transforming growth factor beta (TGF-β) and also suppressed signal transducer and activator of transcription 3 (STAT-3) expressions and STAT3-phosphorylation. The blocked STAT3-phosphorylation in T cells also blocked the differentiation of Th-17 cells (Xie et al., 2009). In addition, the role of curcumin in the pathogenesis of multiple sclerosis has also been investigated. The results showed that curcumin inhibited the toll-like receptors 4 (TLR4) and toll like receptors 9 (TLR9) expression on CD4+ T and CD8+ T cells, immunized with PLPp139-151 and MOGp35-55 antigen. Mechanistically, it was found that these toll like receptors acted as co stimulatory receptors. Curcumin treatment led to the decrease in the expression of PLPp139-151 and MOGp35-55 Ag-induced TLR4 and TLR9 on the CD4+ T cells and CD8+ T cells, which also ameliorated this disease. It was found that TLR 4 and TLR9 acted as co-stimulatory receptors which further boost the cytokine production and proliferation in response to the specific agonists (Chearwae and Bright, 2008). Earlier, it was narrated that the inhibitory abilities of curcumin on IL-12 production in spleen T lymphocytes ameliorated experimental autoimmune encephalomyelitis (Natarajan and Bright, 2002). In a model of type II collagen induced arthritis (CIA), curcumin abrogated lymphocytes proliferation induced by bovine type II collagen Ag (Moon et al., 2010). And also found to inhibit PMA induced proliferation of T cells isolated from human spleen (Jagetia and Aggarwal, 2007). Curcumin exerted its immunosuppressive effects on T cells by tyrosine kinase, a CD28 co-stimulatory pathway of T-cell activation (Ranjan et al., 2004). Peripheral blood mononuclear cells (PBMCs) are largely comprised of T cells. Maier et al. documented that curcumin caused dose-dependent suppression of TH-1 type immune responses in mitogen stimulated PBMCs (Maier et al., 2010). Mechanistically, it was shown that this suppressive effect accounts to the inhibition of neopterin production mediated by IFN-γ along with the degradation of tryptophan.

### Immunosuppressive effects of zerumbone on T cells

Abdelwahab et al. (2011) experimented the effects of zerumbone on T-acute lymphoblastic leukemia, CEM-ss cancer cells. Zerumbone was found cytotoxic to CEM-ss cells with an IC_50_ value of 8.4 ± 1.9 μg/mL. Additionally, zerumbone enhanced the number of TUNEL-positive stain and cellular level of caspase-3 on CEM-ss cells. Therefore, it's postulated that zerumbone has the ability to induce apoptosis on T-acute lymphoblastic leukemia, CEM-ss cells as well as it may help to cure leukemia. Similarly, another study was conducted to check the effect of zerumbone loaded into nanostructured lipid carriers (ZER-NLC) in human T-cell acute lymphoblastic leukemia (Jurkat) cells and concluded that ZER-NLC were able to possess antileukemic as a sustained-release drug carrier system for treating leukemia (Rahman et al., 2013). The author also reported the antiproliferative effect of zerumbone on T- cells (Jurkat) through apoptotic intrinsic pathway followed by activating caspase-3 and caspase-9 (Rahman et al., 2014).

### Immunosuppressive effects of chalcones and derivatives on T cells

Chalcones with the reactive α,β-unsaturated carbonyl group have also been found to abrogate the production and function of normal cytotoxic (CD8) T cells in mouse spleen (Schwartz and Middleton, 1984). In contrast, another study documented that chalcone isoliquiritigenin **(74)** exhibited no effect on proliferation of lymphocytes induced by mitogen Con A and also mixed lymphocyte culture from mouse spleen (Namgoong et al., 1994). The chemical structure of compound **74** is shown in Figure 5. The antiproliferative effects of xanthohumol **(73)** as mentioned earlier were also investigated on T cells. It was reported that xanthohumol significantly inhibited the proliferation and development of cytotoxic T cells along with the release of cytokines from helper T cells (Th1) i.e., IL-2, IFN-γ, and TNF-α. These inhibitory effects were found to be associated with the depletion of IκBα phosphorylation in the NF-κB signaling pathway (Gao et al., 2009). In contrast, Choi et al. (2009) demonstrated that xanthohumol enhanced the release of cytokine IL-2 in a dose dependent fashion from PMA and ionomycin activated mouse EL-4 T cells. These elevated effects were attributed to the upregulation of nuclear factor of activated T cells and AP-1 in mouse EL-4 T cells.

Barfod et al. (2002) reported that the proliferation of T cells as well as release of IFN-γ from PHA-stimulated T cells has been dose dependently inhibited by licochalcone A, extracted from Chinese liquorice. Similarly another chalcone, naringenin **(60)** was investigated against helper T cells (CD4+) sensitized by OVA and it was observed that this chalcone notably reduced the secretion of Th2 cytokines i.e., IL-4, IL-5, and IL-13, from mice splenic CD4T cells (Iwamura et al., 2010). Naringenin, found in grapefruit and other citrus fruits, significantly abrogated picryl chloride (PCl)-induced contact hypersensitivity by suppressing the proliferation and activation of T lymphocytes. The mouse T cells were activated by anti-CD3 and anti-CD28. In addition, the aforementioned chalcone also inhibited the mRNA expressions of different cytokines, including IL-2, IFN-γ, and TNF-α. Simultaneously, naringenin also showed T cell apoptosis which was subjected to the upregulation of Bax, Bad, PARP (poly ADP ribose polymerase), cleaved-caspase 3 while downregulation of phosphorylated Akt, Bcl-2 was observed (Fang et al., 2010).

### Immunosuppressive effects of curcumin on B cells

A study conducted by Han et al. (1999) showed that curcumin exerted antiproliferative effects in a dose-dependent manner on BKS-2, an immature B cell lymphoma. It was suggested that curcumin inhibited the proliferation and induced apoptosis of BKS-2 by inhibiting the activity of NF-κB and further suppressing the expression of survival and growth genes i.e., early growth response protein (egr-1), regulator gene that codes for transcription factor, (c-myc) Bcl-xL, tumor suppressor gene p53 (Han et al., 1999). Another study demonstrated that curcumin was a potent inhibitor of B cell activation and also associated signaling pathways. It was found that curcumin treatment demolished proliferation of B cells stimulated by toll-like receptor ligands LPS and CpG oligodeoxynucleotides. Furthermore, these inhibitory effects were accounted for the inhibition of phosphorylation of ERK, Iκ-B, and p38 kinase, NF-κB activation (Decoté-Ricardo et al., 2009). These observations indicated the anti-inflammatory effects of curcumin in the B cell response. Likewise, curcumin exerted immunosuppressive effects by inhibiting not only LPS induced proliferation of B cells in a dose-dependent manner but also LPS-induced secretion of IgG1 and IgG2 antibodies. The underlying mechanisms indicated were the downregulation of CD28 and CD80 expression along with up-regulation of CTLA-4 (Sharma et al., 2007). Correspondingly, the inhibitory effects of curcumin on production of IgE antibodies from rat splenocytes were also evaluated (Kuramoto et al., 1996).

Interestingly, in one study, curcumin has also been found to impede the B cell immortalization isolated from healthy human volunteers and stimulated by Epstein barr virus (EBV). This process of immortalization led to post-transplant lymphoproliferative disorders in patient usually on immunosuppressive drugs like cyclosporine A. However, the mechanism behind this effect was not explained (McDonald et al., 1998). Subsequently another study explained that curcumin regulated the B cell immortalization effect by causing an increased apoptosis in the B cells infected by EBV (Ranjan et al., 1999). Curcumin significantly elevated the production of IgG antibodies from B cells at a higher dose of 40 mg/kg as compared to lower doses (1, 20 mg/kg) where the antibody levels were found similar as that of control (South et al., 1997). Curcumin can also play a crucial role in the treatment of B cell autoimmune diseases. As evidence from one study, curcumin has shown concentration-dependent inhibition of activation-induced cytosine deaminase (AID) expression, an enzyme regulated by NF-κB and considered important for Ig class switch recombination and somatic hyper-mutation having a role in tumorigenesis. This effect was subjected to the demolished recovery of IgG+ class-switched B cells (Haque et al., 2010).

### Immunosuppressive effects of curcumin on natural killer cells

A number of studies have investigated the role of α,β-unsaturated carbonyl-based compounds on natural killer cell activity against tumor cells. Earlier studies reported that administration of different doses of curcumin for duration of 5 weeks exhibited no effect on NK cell activity (South et al., 1997). Correspondingly, another study described that *in vivo* curcumin treatment for long duration exerted no effect on NK cell activity in ascites tumor development (Varalakshmi et al., 2008). In contrast, Yadav et al. (2005) demonstrated that treatment with curcumin enhanced the *in vitro* cytotoxicity of NK cells which could be supplemented by IFN-γ treatment. Lymphokine activated killer cells (LAK) are analogous to natural killer cells. Gao et al. (2004) evaluated the production of these non-specific LAK cells induced by IL-2 in addition to the determination of cytotoxic activity against YAC-1 lymphoma cells. The results concluded that higher doses of curcumin significantly reduced LAK cell production. It was also documented that curcumin treated natural killer cells, isolated from healthy donors, suppressed the release of IFN-γ and granzyme B in the presence of K562 and A375 melanoma cell lines (Bill et al., 2009). Recently, curcumin has also been found to increase the degradation of tumor derived exosomal proteins which ultimately inhibited the NK cell activity induced by IL2 against breast carcinoma. These effects of curcumin led to anti-tumor immune responses (Zhang et al., 2007). Another study displayed that curcumin exerted cytocidal effects by causing the cell death of NKL, NK-92, and HANKI (NK/T-cell lymphoma cell lines) which were not responding to other therapies. This anti-proliferative activity was subjected to the inhibition of NF-κB activation in addition to suppression of Bcl-xL, cyclin D1, XIAP, and c-FLIP expression and the subsequent cleavage and activation of caspase-8 and poly (ADP-ribose) polymerase (Yadav et al., 2005). Likewise, the inhibited proliferation of LAK cells was studied (Mandal and Viswanathan, 2015).

### Immunosuppressive effects of chalcone and its derivatives on natural killer cells

Flavokawain A chalcone depicted *in vivo* antitumor activity in a dose-dependent manner and ultimately inhibiting NK cell activity in mice via interferon-a synthesis induction (Hornung et al., 1988). Wleklik et al. (1988) suggested that mice treated with amentoflavone or quercetin developed measurable serum content of interferon. The antitumor activity of naturally occurring flavonoids could be attributable to the immunomodulatory properties of induced interferons with associated augmentation of NK cell function (Verma et al., 1988). NK cell cytocidal activity against NK-sensitive K562 and U937 tumor target cells was accompanied by early increased incorporation of 32P into PI, suggesting activation of phospholipase C (Steele et al., 1990).

## Conclusion and future prospects

α, β-Unsaturated carbonyl groups are widely distributed as reactive substructures of natural compounds and this has been exemplified by the natural products, curcumin, chalcone, and zerumbone. The immunosuppressive effects of these α,β-unsaturated carbonyl-based compounds and their analogs and derivatives have been comprehensively studied on neutrophils, monocytes, and macrophages. These compounds have also been investigated on other types of immune cells i.e., T cells, B cells, natural killer cells, and dendritic cells but far less as compared to neutrophils and macrophages. It is very important to further explore the biological reactivity of α, β-unsaturated carbonyl groups, especially on their effects on the immune cells in different pathological states. The inhibitory effects of the α, β-unsaturated carbonyl-based compounds on the immune cells play key role in inflammation. The anti-inflammatory activity of the compounds were partially mediated by their effects on the immune cells specifically on inhibition of pro-inflammatory cytokines expression, NO production, NF-κB activation, and iNOS. This review may provide useful information for other scientists to further examine more pharmacological effects of α, β-unsaturated carbonyl-compounds in different types of immune cells and hence come up with a promising agent that may serve as a lead compound for further development into potent immunomodulating agent in treating different immune related diseases particularly related to inflammatory disorders.

## Author contributions

IJ and SNAB participated in the concept and editing of the manuscript and IJ gave the final approval of the final version to be submitted for publication. LA and MAH drafted the manuscript.

### Conflict of interest statement

The authors declare that the research was conducted in the absence of any commercial or financial relationships that could be construed as a potential conflict of interest.

## Abbreviations

APC: antigen presenting cells
NF-κB: nuclear factor kappa B
TNFα: tumor necrosis factor alpha
ROS: reactive oxygen species
IL: interleukin
PBMCs: peripheral blood mononuclear cells
PMNCs: polymorphonuclear cells
fMLP: N-Formylmethionine-leucyl-phenylalanine
PKC: protein kinase C
ICAM-1: Intercellular Adhesion Molecule 1
VCAM-1: Vascular cell adhesion protein 1
CINC: cytokine induced neutrophil chemoattractant
OVA: ovalbumin
iNOS: inducible nitric oxide synthase
NO: nitric oxide
LTB4: leukotriene B4
PMA: phorbol 12-myristate 13-acetate
LPS: lipopolysaccharide
UVB: ultraviolet-B
BALF: bronchoalveolar lavage fluid
CON A: concanavalin A
ERK: extracellular signal-regulated kinases
MCP-1: Monocyte chemoattractant protein-1
MIP-1β: Macrophage Inflammatory Protein 1-beta
CCR5: chemokine receptor 5
AChE: acetylcholinesterase
PLA2: phospholipase A2
PGE2: prostaglandin E2
COX 2: cyclooxygenase 2
LOX: lipoxygenase
IFN-γ: interferon gamma
AP-1: activated protein 1
TLR: toll like receptors
DCs: dendritic cells
MHC: major histocompatibility complex
PHA: Phytohaemagglutinin
STAT: Signal transducer and activator of transcription 3
LAK: lymphokine activated killer cells.
